# Environmental enrichment has no effect on the development of dopaminergic and GABAergic fibers during methylphenidate treatment of early traumatized gerbils

**DOI:** 10.1186/1477-5751-7-2

**Published:** 2008-05-16

**Authors:** Susanne Brummelte, Thorsten Grund, Gunther H Moll, Gertraud Teuchert-Noodt, Ralph R Dawirs

**Affiliations:** 1 Department of Neuroanatomy/Cognitive Neuroscience, Faculty of Biology, University of Bielefeld, Universitätsstrasse 25, D-33615 Bielefeld, Germany; 2 Department of Child and Adolescent Psychiatry, University Hospital Erlangen, Schwabachanlage 6 + 10, D-91054 Erlangen, Germany; 3 Department of Psychology, University of British Columbia, 2136 West Mall, Vancouver, BC, V6T 1Z4, Canada

## Abstract

It is widely believed, that environmental factors play a crucial role in the etiology and outcome of psychiatric diseases such as Attention-Deficit/Hyperactivity Disorder (ADHD). A former study from our laboratory has shown that both methylphenidate (MP) and handling have a positive effect on the dopaminergic fiber density in the prefrontal cortex (PFC) of early traumatized gerbils (*Meriones unguiculatus*). The current study was performed to investigate if enriched environment during MP application has an additional influence on the dopaminergic and GABAergic fiber densities in the PFC and amygdala in this animal model.

Animals received a single early dose of methamphetamine (MA; 50 mg/kg; i.p.) on postnatal day (PD) 14, which is known to cause multiple changes in the subsequent development of several neurotransmitter systems including the dopaminergic systems, and were then treated with oral daily applications of MP (5 mg/kg) from PD30–60. Animals treated this way were either transferred to an enriched environment after weaning (on PD30) or were kept under impoverished rearing conditions.

There was no effect of an enriched environment on the dopaminergic or GABAergic fiber density neither in the PFC nor in the amygdala. With regard to former studies these results underline the particular impact of MP in the treatment of ADHD.

## Findings

Methylphenidate (MP) (e.g. Ritalin^®^) is a stimulant drug and is the common medicament to treat Attention-Deficit/Hyperactivity Disorder (ADHD) as it is reducing the core symptoms of this frequent adolescent disease [1,2]. Being an indirect dopamine (DA) agonist MP blocks the reuptake of DA through the DA transporter and the noradrenalin transporter [3-5], and thus leads to an increased extracellular concentration of DA [6,7]. The neurobiological basis of ADHD is basically thought to be characterized by deficient dopaminergic systems [8,9], with meso-limbo-cortical and nigro-striatal dopaminergic structures being differentially affected [10-12].

**Figure 1:**
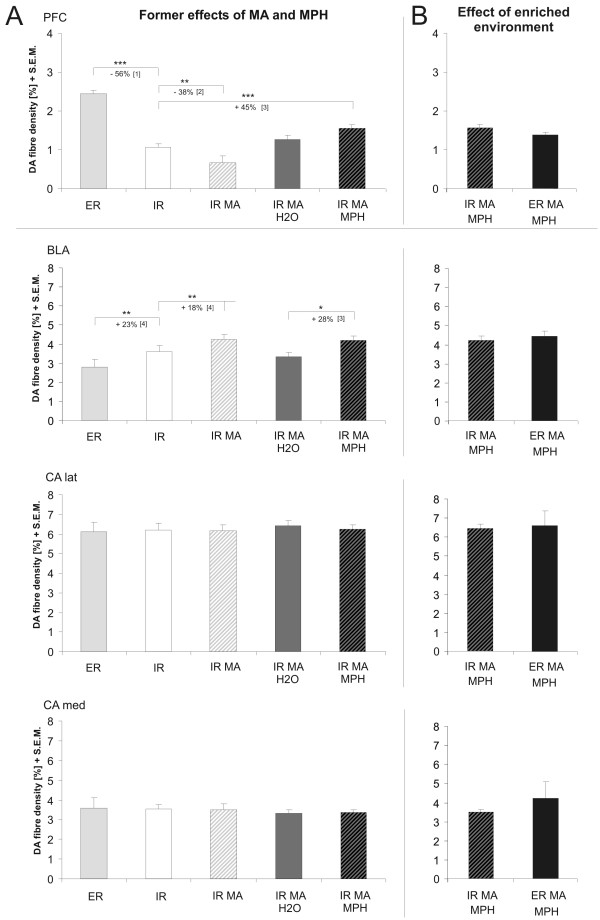
A. Overview over previously published effects of methamphetamine (MA) and methylphenidate (MP) on the dopaminergic fiber densities in the amygdala and the medial prefrontal cortex (PFC) of animals from enriched (ER) an impoverished rearing (IR) conditions. Values were nominated to account for possible variations in the data due to different experimenters and procedures to make them comparable. B. Effect of transfer to enriched environment on the dopaminergic fiber density. There was no significant effect in any of the investigated areas. (Therefore data from different laminae of the PFC was combined here). Abbr.: DA: Dopamine; ER. Enriched environment; IR: Impoverished environment; MA: Methamphetamine, MP: Methylphenidate; BLA: Basolateral Amygdala; CA lat. Lateral part of the central amygdala; CA med: Medial part of the central amygdala; PFC: Prefrontal cortex; [1]: Winterfeld et al., 1998; [2]: Dawirs et al., 1994; [3]: Grund et al., 2006; [4]: Busche et al. 2004.

Our lab has studied the long-term plastic effects of methylphenidate (MP) in an animal model of early traumatization, that bears some resemblance to ADHD [13,14]. We challenged gerbils (*Meriones unguiculatus*) with a single non-invasive dose of methamphetamine (MA, 50 mg/kg, i.p.) on postnatal day 14 [15], which causes an imbalance in the dopaminergic system, in particular a reduced DA fiber density in the prefrontal cortex and the nucleus accumbens and an increased dopaminergic innervation in caudal limbic areas [16-18]. The oral application of MP for 30 days to those previously traumatized gerbils, leads to an increase in prefrontal dopaminergic fiber densities compared to controls, which received H_2_O instead [14], thus restoring pristine fiber densities in non-traumatized gerbils (see Fig. 1). However, the fiber densities in the nucleus accumbens and in the amygdala were not or only slightly affected, underlining a rather specific effect of MP[13,14].

Parental care and family environment have been linked to ADHD [19], as e.g. revealed by an association between low social status, early deprivation or high family conflict and ADHD [19-22]. Recent results from an animal study further suggest an association between maternal stress during the postpartum period and hyperactive and impulsive behavior, particularly in the male offspring [23]. However, a good environment has been shown to positively affect the development of young children from adverse family situations [20], underlining the importance of taking social and family milieu into account concerning the diagnosis and treatment of hyperactive children.

The current study was performed to investigate the potentially augmenting effect of an enriched environment on the impact of MP, measured by dopaminergic and GABA fiber densities. The GABAergic and dopaminergic systems are known to exhibit a high interconnectivity with e.g. DA innervating GABAergic cell bodies, dendrites and axon terminals in either a excitatory or inhibitory way [24-26]. As it has further been shown that the GABAergic system reacts with particular changes in its local innervation pattern to an early disturbance of the dopaminergic system [27,28], it is conceivable that GABAergic elements play an essential role in reactive neuroplasticity [29]. Therefore, this transmitter system was additionally investigated to reveal potential adaptation or compensations within the local networks of altered dopaminergic terminal inputs.

Breeding gerbils (*Meriones unguiculatus*) were obtained from Harlan Winkelmann (Borchen, Germany) and kept under natural day/night cycles with food and water being provided *ad libitum*. Animals were bred in standard cages (Makrolon type 4) and received a single non-invasive injection of (+)-methamphetamine hydrochloride (MA) (Sigma, M 8750; 50 mg/kg, i.p.), on postnatal day 14, causing an imbalance in the dopaminergic system [16,17]. On postnatal day (PD) 30, animals were weaned and randomly assigned to one of the two following groups: group 1 (n = 9) was kept individually in standard cages (Makrolon type 3) under impoverished rearing conditions (IR), while group 2 (n = 11) were transferred with their siblings to large compounds (1 m × 1 m) with an environment consisting of opportunities to hide and play and thus kept under semi-natural enriched rearing conditions (ER). All gerbils received an oral daily application of MP (5 mg/kg; Ritalin^® ^IR, Novartis Pharma GmbH, Nürnberg) from PD30–PD60, which appears to properly simulate clinically relevant treatment [cf. [14]]. MP was administered directly through a pipette, rather than given through the drinking water as gerbils do not use to drink regularly. All experimental procedures were approved by the appropriate committee for animal care in accordance with the European Communities Council Directive and all efforts were made to minimize animal number and suffering.

At PD90 all animals were transcardially perfused under deep chloral hydrate anesthesia (1.7 g/kg, i.p.) with 0.1 M sodium cacodylate pH 6.2, followed by 5% glutaraldehyde in 0.1 M sodium cacodylate pH 7.5. Immediately after perfusion the brains were dissected and 50 μm thick frontal sections of the right hemisphere were cut with a vibratome (Leica VT 1000S). The methods used for DA and GABA immunohistochemistry have been published recently [27,30]. DA fibers were quantified in different laminae (I, III) of the prefrontal cortex (PFC), in the basolateral amygdala (BLA) and the medial and lateral part of the central amygdala (CA), while GABA fibers were only investigated in the areas were MP has been shown to affect DA fibers before, namely in the PFC (lamina I, II, III and V/VI) and the BLA (anterior, posterior). In the defined region of each section all detectable fiber fragments were visualized using a bright field microscope (BX61, Olympus, Hamburg, Germany) and a digital camera for microscopy (ColorView II, SIS, Münster, Germany). Fibers were quantified by software for image analysis (KS300, Jenoptik, Jena, Germany) and the fiber density was computed as a percentage of the evaluated test area. Fiber densities were analyzed separately for each region with a repeated-measures analysis of variance (ANOVA) with area (subregions; layers) as within-subject factor and rearing condition (impoverished/enriched) as between-subject factors. All calculations were performed using Statistica 6 (StaSoft, Tulsa USA) with significance level set at p < 0.05 (*).

The repeated measures ANOVA revealed no significant effect of rearing conditions (F (1,17) = 0.50, p = 0.49) or interaction effect of condition and lamina (F (1,17) = 0.07, p = 0.79) for the DA fibers in the prefrontal cortex, or for the DA fibers in the amygdaloid complex (effect of rearing conditions: F (1,16) = 1.32, p = 0.27; interaction effect of condition and area: F (2,32) = 0.8, p = 0.46). The GABA fibers also revealed no significant effect for the environmental condition (PFC: F (1,14) = 0.89, p = 0.36; BLA: F (1,14) = 0.55, p = 0.47), nor was there any interaction effect of condition and lamina/area (PFC: F (3,42) = .30, p = 0.82; BLA: F (1,14) = 3.73, p = 0.073).

So apparently enriched environment has no augmenting effect on the action of MP on the fiber system of DA (Fig. 1), nor were there any adaptive changes in the GABAergic system in the prefrontal cortex or amygdala of afore traumatized gerbils.

There are to date only few studies, which have investigated the long term effects of MP on the developing brain. Moll and colleagues [31] could show that the dopamine transporter density was reduced in rat striatum after early exposure to a clinical dose of MP. In addition, our lab could recently reveal an increase in dopaminergic fiber density in the medial PFC and the BLA of MP treated gerbils which were traumatized by the psychostimulant drug MA [[13]; cf. Fig. 1]. This MA-traumatization, which was also used in the current study, is particularly effective to cause disturbances in the developing rather than in the adult brain [32]. Therefore, the early pharmacological challenge in our animal model is used to create a specific pathological state in the dopaminergic system [16,17] that is likely to mimic behavioral and neuroanatomical aspects of ADHD [13].

Further long-term effects should also be expected from environmental variables, as these are usually thought to play a potentially important role in the modulation of ADHD [20,33-35]. In fact, enriched rearing of animals has been shown to attenuate behavioral changes after brain injuries [36] and improve cognitive functions [37-39]. Therefore, treating ADHD children usually includes a combination of drug treatment and behavioral interventions [34,40,41], although controlled clinical trials gave rise to controversial discussions about the augmenting impact of behavior therapy [34].

Considering the latter it is less surprising that we could not detect any structural improvement in animals, which were transferred from impoverished rearing conditions to an enriched environment while receiving MP. On the other hand, a former study has already revealed an effect of handling on the dopaminergic system without any medication [13]. Interestingly, this handling effect was only significant in saline treated animals and not in MA-treated animals compared to unhandled controls. As animals from impoverished rearing conditions already reveal an altered innervation density of different transmitter systems compared to animals born and reared in semi-natural environment [[17,39,42-44]; cf. Fig. 1], this handling effect may be interpreted as a beneficial "therapeutic" intervention [13]. This hypothesis is supported by a study showing, that the increase in extracellular dopamine in the mPFC after MP exposure is significantly elevated when combined with handling [45]. Thus, it is conceivable, that the transfer to an enriched environment has no additional positive consequences. Although most studies so far have concentrated on neonatal handling (for review see [46]) rather than on handling after weaning, the procedure clearly excite functional neuronal adaptations within cortical and endocrine systems [47,48]. It would be interesting to investigate the effect of MP during environmental enrichment without the additional handling, however, due to the group housing and irregular drinking habits of gerbils administration of MP through the drinking water is not a suitable alternative.

The late transfer to the enriched environment in our study might be an additional reason for the failure of this intervention to reveal any differences. Therefore, a functional improvement in the transmitter systems might be possible by environmental enrichment but structural changes might be sensitive to critical phases or the beneficial impact might be obscured by the dominating effects of MP or the impact of handling.

## List of Abbreviations

ADHD: Attention-Deficit/Hyperactivity Disorder; MP: methylphenidate; PFC: prefrontal cortex; MA: methamphetamine; DA: dopamine; BLA: basolateral amygdala; CA: central amygdala

## Competing interests

The authors declare that they have no competing interests.

## Authors' contributions

SB participated in the acquisition and interpretation of the data and drafted the manuscript.

TG contributed to the acquisition and the analysis of the data

GM contributed to the study conception design and interpretation of data.

GT participated in the design of the study, and the drafting and revision of the manuscript.

RD participated in the design of the study and the critical reviewing of the manuscript.

All the authors have read and approved the final manuscript.
